# Changes in carpal tunnel compliance with incremental flexor retinaculum release

**DOI:** 10.1186/s13018-016-0380-3

**Published:** 2016-04-13

**Authors:** Rubina Ratnaparkhi, Kaihua Xiu, Xin Guo, Zong-Ming Li

**Affiliations:** Hand Research Laboratory, Departments of Biomedical Engineering, Orthopaedic Surgery, and Physical Medicine and Rehabilitation, Cleveland Clinic, 9500 Euclid Avenue, Cleveland, 44195 OH USA

**Keywords:** Carpal tunnel release, Transverse carpal ligament, Distal aponeurosis, Antebrachial fascia, Compliance

## Abstract

**Background:**

Flexor retinaculum transection is a routine surgical treatment for carpal tunnel syndrome, yet the biomechanical and clinical sequelae of the procedure remain unclear. We investigated the effects of flexor retinaculum release on carpal tunnel structural compliance using cadaveric hands.

**Methods:**

The flexor retinaculum was incrementally and sequentially released with transections of 25, 50, 75, and 100 % of the transverse carpal ligament, followed by the distal aponeurosis and then the antebrachial fascia. Paired outward 10 N forces were applied to the insertion sites of the transverse carpal ligament at the distal (hamate-trapezium) and proximal (pisiform-scaphoid) levels of the carpal tunnel. Carpal tunnel compliance was defined as the change in carpal arch width normalized to the constant 10 N force.

**Results:**

With the flexor retinaculum intact, carpal tunnel compliance at the proximal level, 0.696 ± 0.128 mm/N, was 13.6 times greater than that at the distal level, 0.056 ± 0.020 mm/N. Complete release of the transverse carpal ligament was required to achieve a significant gain in compliance at the distal level (*p* < 0.05). Subsequent release of the distal aponeurosis resulted in an appreciable additional increase in compliance (43.0 %, *p* = 0.052) at the distal level, but a minimal increase (1.7 %, *p* = 0.987) at the proximal level. Complete flexor retinaculum release provided a significant gain in compliance relative to transverse carpal ligament release alone at both proximal and distal levels (*p* < 0.05).

**Conclusions:**

Overall, complete flexor retinaculum release increased proximal compliance by 52 % and distal compliance by 332 %. The increase in carpal tunnel compliance with complete flexor retinaculum release helps explain the benefit of carpal tunnel release surgery for patients with carpal tunnel syndrome.

## Background

The flexor retinaculum (FR) consists of three continuous but distinct segments from the proximal to distal: the antebrachial fascia (AF), transverse carpal ligament (TCL), and distal aponeurosis (DA) [1, 2]. The AF is a membranous tissue that provides distal reinforcement of the volar antebrachial fascia [3]. The TCL is a dense transverse fibrous lamina defined by its attachments to the pisiform and the hook of the hamate medially and the tubercles of the scaphoid and the trapezium laterally. The DA is a fibrous septum between the bases of the thenar and hypothenar muscles [3].

Carpal tunnel release represents the gold-standard surgical treatment for carpal tunnel syndrome, a painful entrapment neuropathy caused by compression of the median nerve within the carpal tunnel. Historically, the terms FR and TCL have both been used in reference to the ligamentous structure transected in carpal tunnel release surgery. However, most commonly, it is primarily the TCL that is released, with varying extents of the AF and DA transection, depending on the surgical technique, the surgeon’s preference, and the wrist involved [2]. Surgical release decompresses the median nerve by increasing the tunnel size and reducing tunnel pressure [4]. Although surgical treatment is considered effective in relieving symptoms in a majority of carpal tunnel syndrome cases, post-operative complications and symptom recurrence are reported in 25 % or more cases [5–7]. Incomplete FR release has been considered a causative mechanism for new, persistent, or recurrent symptoms that may require later revision surgery to improve inadequate median nerve decompression stemming from residual focal mechanical restraints [4, 8]. However, the FR plays an important role in carpal tunnel mechanics, and carpal tunnel release unavoidably affects the structural integrity and flexibility of the carpal tunnel [9]. There is lingering debate about whether transection of all three FR segments is required to adequately release the median nerve, given the potential for undesirable complications with either incomplete or excessive FR release.

Previous research has examined the morphological changes associated with FR release as a means of understanding the procedure’s effects on the structural integrity and flexibility of the carpal arch. In vivo and in vitro studies have demonstrated that carpal tunnel release leads to increases in carpal tunnel volume and in the cross-sectional and carpal arch areas [10–14]. In contrast, the effect of FR release on carpal arch width remains inconclusive, as both an increase and no change in carpal arch width have been reported in previous studies evaluating FR release depending on the hand positioning and imaging modality used [10, 11, 15]. In vitro studies completed under well-controlled experimental conditions have shown that carpal tunnel release drastically affects the biomechanical properties of the carpal tunnel [13, 16, 17]. For example, Tengrootenhuysen et al. [17], who applied constant loads to the carpal tunnel, demonstrated that the carpal arch was increasingly extensible with incremental release of the TCL. Xiu et al. [16] showed that complete TCL release led to a location-dependent increase in carpal arch compliance, with a greater relative increase in compliance at the distal than at the proximal carpal tunnel. Kim et al. [14] reported that complete TCL release caused a ninefold increase in the compliance of the carpal arch, as quantified by the pressure-area relationship. However, it remains unclear how different degrees of FR release, including both intermediate transection of increasing percentages of the TCL and complete transections of the DA and AF, alter the compliance of the carpal arch at both the proximal and distal levels.

The purpose of this study was to investigate the effect of incremental FR release on the structural mechanics of the carpal tunnel, focusing on the compliance related to carpal arch width at multiple locations in the carpal tunnel. We hypothesized that carpal tunnel compliance would progressively increase with incremental FR release and that the impact of FR release would differ between the proximal and distal levels.

## Methods

### Specimens and preparation

**Fig. 1:**
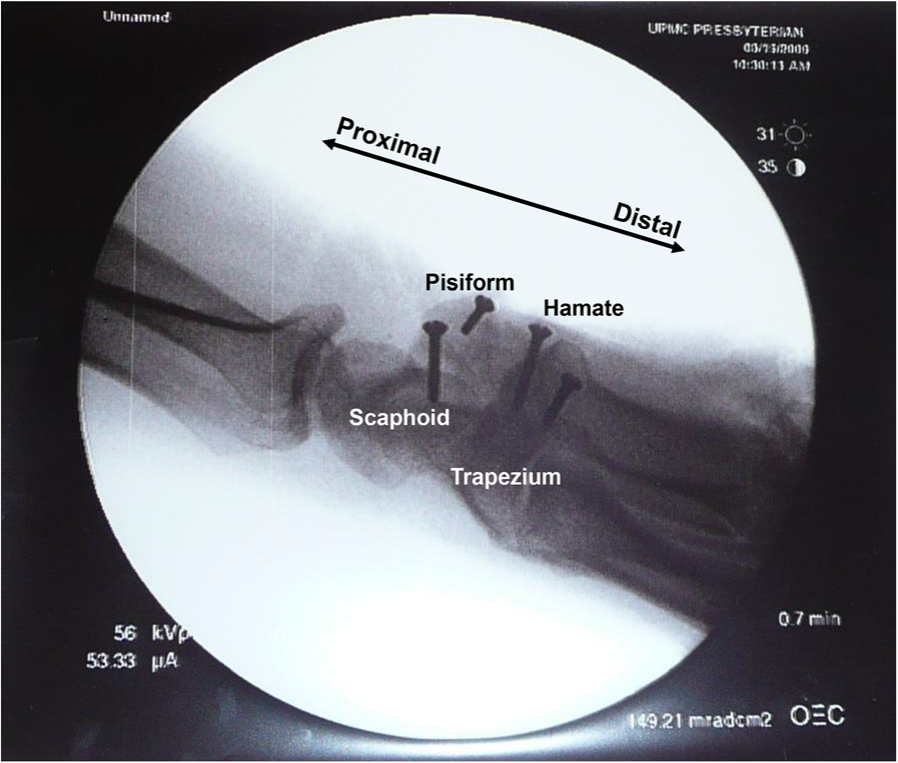
Lateral X-ray image showing intra-experimental cortical screw insertion onto the carpal bones of a cadaver hand. These screws served as the points of application of the paired outward loading forces (10 N) at the proximal and distal levels of the carpal tunnel

Nine fresh frozen cadaver hands (five men and four women; mean age, 50 ± 11 years) were used in this study. The cadaveric hands were obtained from Anatomy Gifts Registry (Hanover, MD), and the experimental use of the cadaveric specimens was approved by Cleveland Clinic Institutional Review Board. All specimens were screened by review of medical records and by X-ray examinations to exclude any specimens with musculoskeletal disorders, traumatic injuries, or arthritic changes in the hands or wrists. The specimens were thawed overnight at room temperature. Dissection was then performed to expose the FR by removing the skin and palmar fascia. The three portions (AF, TCL, and DA) of the FR were identified. Then, cortical screws (Synthes, Inc., West Chester, PA), 2.0 mm in diameter, were drilled into the hamate, trapezium, pisiform, and scaphoid at the sites of TCL insertion until the screw head was flush with the bony surface (Fig. 1). A thin wire was attached to each screw head to assist in force application.

### Apparatus design and specimen alignment

A custom apparatus was developed in our laboratory to affix each hand specimen and apply the prescribed force [16]. The apparatus included a platform with a wedge-shaped plywood block to stabilize the specimen, with adjustable pulley systems for aligning applied forces. The wrist of each specimen was positioned in a 20° extension by aligning the dorsal side of the hand specimen with the surface of the wedge-shaped plywood block on the platform. The specimen was then affixed by drilling a screw through the middle shaft of the third metacarpal into the block. Velcro strips were used to strap the forearm to the base block for further stabilization. A T-slotted track with two sliders was configured at each of the two parallel edges of the platform along the proximal-distal direction of the hand. Each slider was attached to a pulley, which was adjustable in both horizontal and vertical directions for force alignment.

### Experimental procedures

**Fig. 2:**
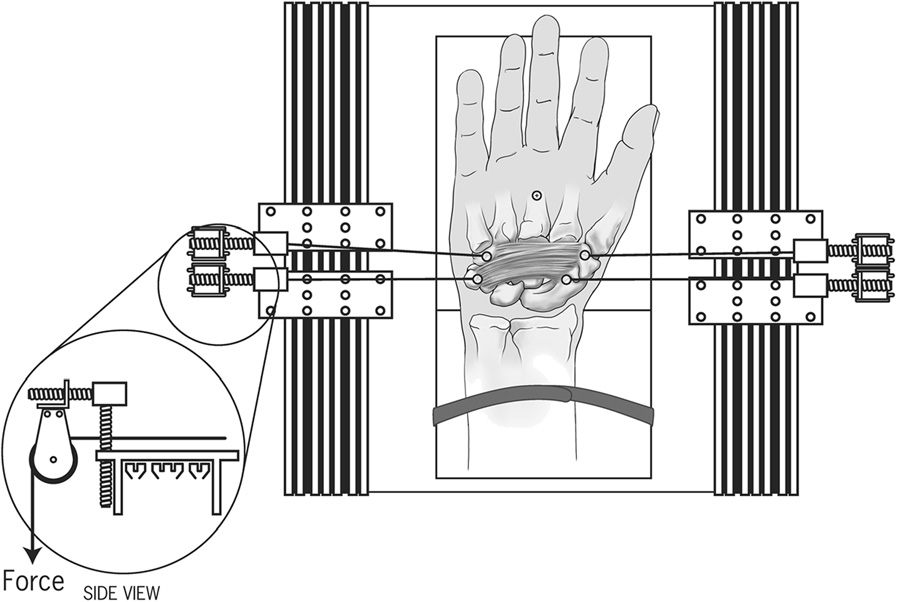
Experimental setup used to simultaneously apply two pairs of outward 10 N loading forces to the carpal bones at the proximal and distal levels

Two pairs of 10 N forces were applied to the bones in the outward direction via the screw and wire system. One force pair was aligned along the line connecting the hamate and trapezium at the distal level of the carpal tunnel, and the other was aligned along the line connecting the pisiform and scaphoid at the proximal level (Fig. 2).

**Fig. 3:**
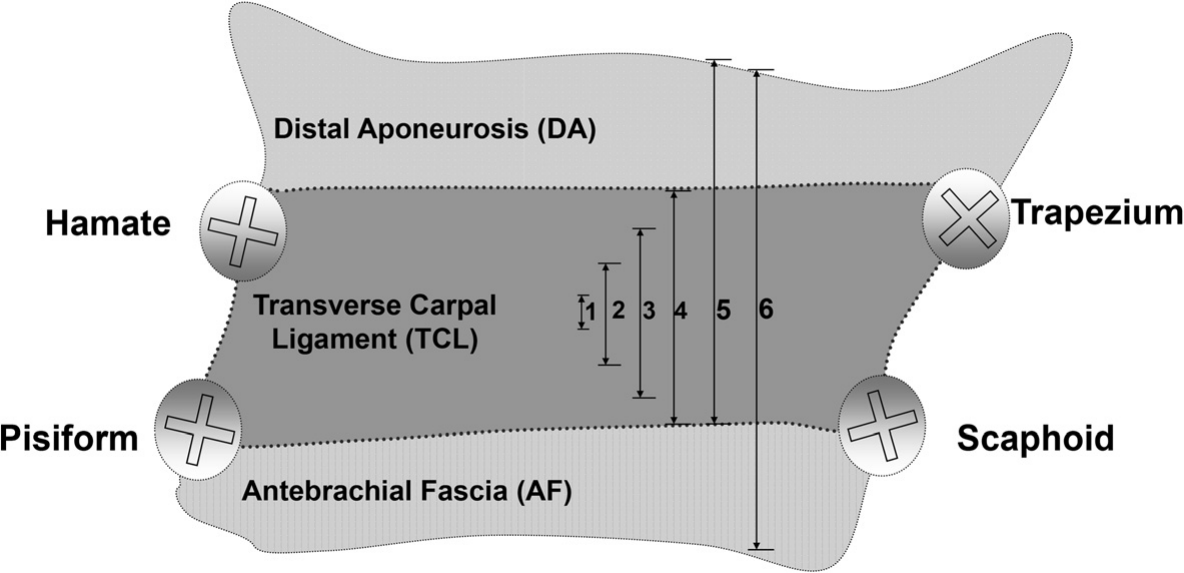
Stepwise release of the FR. *(1)* 25 % TCL release, *(2)* 50 % TCL release, *(3)* 75 % TCL release, *(4)* 100 % TCL release, *(5)* 100 % TCL release + DA release and *(6)* 100 % TCL release + DA + AF release. Note that all release steps were completed in the same plane of sectioning. *FR* flexor retinaculum; *TCL* transverse carpal ligament; *DA* distal aponeurosis; *AF* antebrachial fascia

The coordinates of the center of each screw head surface were digitized using a MicroScribe 3D digitizer (Immersion Corp., San Jose, CA) during unloaded and loaded conditions with the FR intact and at each step of FR release. The FR was incrementally released in the following steps: (1) 25 % TCL, (2) 50 % TCL, (3) 75 % TCL, (4) 100 % TCL, (5) 100 % TCL + DA, and (6) 100 % TCL + DA + AF (i.e., complete release of the FR). Incremental FR release started from the center of the TCL, progressed symmetrically to the distal and proximal edge of the TCL, and then the DA and AF were sequentially transected (Fig. 3). After each release step, there was a 1-min unloading period to allow the carpal tunnel structure to recover and stabilize.

### Data analysis

Carpal tunnel compliance was used to quantify structural flexibility and was calculated as the ratio of the load-induced change in carpal arch width to the applied load (i.e., a constant 10 N at each level). Compliance was individually defined for the distal and proximal tunnel for each of the intact and released conditions. Digitized coordinates from the bony landmarks were used to calculate carpal arch width at the distal tunnel between the hamate and trapezium and at the proximal tunnel between the pisiform and scaphoid. Carpal tunnel compliance was defined as (*W* − *W*_0_)/Load, where *W* is the distal or proximal carpal arch width under loading, and *W*_0_ is the initial distal or proximal carpal arch width without loading. Two-way repeated-measures ANOVA (6 × 2) was performed to investigate the effects of the FR release steps and carpal tunnel levels on the carpal tunnel compliance. Post hoc Tukey’s tests were used for pairwise comparisons. All statistical analyses were performed using SigmaStat 3.4 (Systat Software, Inc., San Jose, CA), and results were considered significant for *p* values <0.05.

## Results

**Fig. 4:**
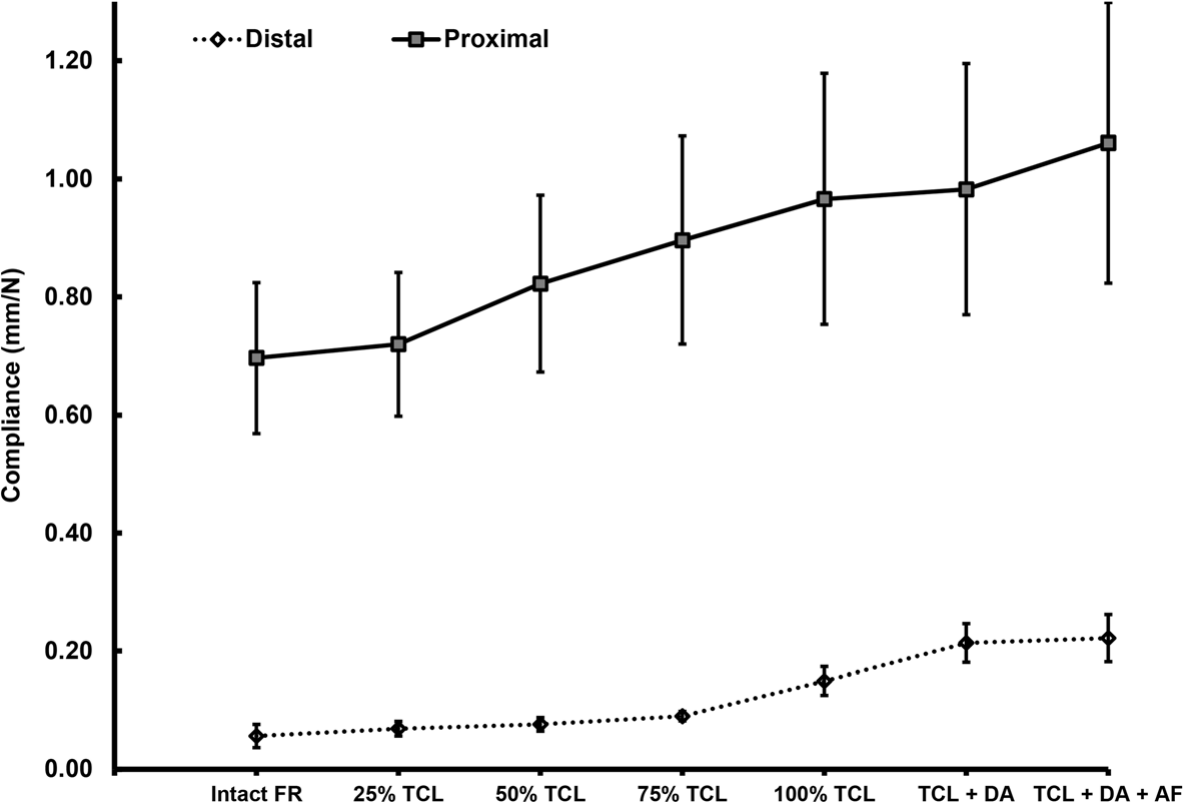
Carpal tunnel compliance as a function of stepwise flexor retinaculum transection at the proximal and distal levels of the carpal tunnel

With an intact FR, carpal tunnel compliance was 0.056 ± 0.020 mm/N at the distal level and 0.696 ± 0.128 mm/N at the proximal level. There was no significant change in compliance observed with 25 % TCL transection at either the distal or proximal carpal tunnel (Fig. 4). The first release step that significantly increased compliance relative to compliance with the FR intact was 100 % TCL release at the distal level and 50 % TCL release at the proximal level (*p* < 0.05). Complete TCL transection led to compliance values of 0.149 ± 0.024 mm/N (distal) and 0.966 ± 0.213 mm/N (proximal). Complete FR release increased compliance relative to the intact condition by 0.166 ± 0.041 mm/N (332 %) at the distal and 0.365 ± 0.137 mm/N (52 %) at the proximal level. The distal tunnel exhibited significantly smaller compliance than the proximal tunnel at every step of FR release (*p* < 0.01). However, FR release progressively reduced the difference in compliance between the proximal and distal levels, from a factor of 13.6 with the FR intact, to a factor of 6.6 with 100 % TCL release, and to a factor of 4.9 with complete FR release.

**Fig. 5:**
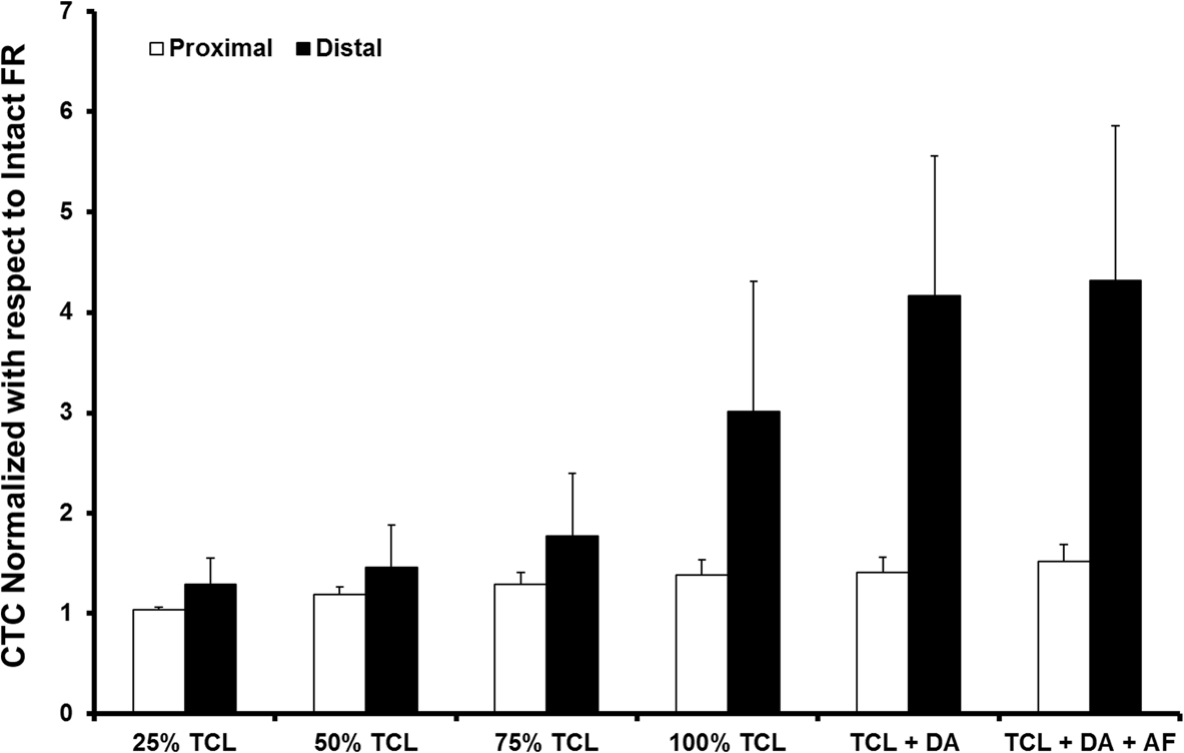
Ratio of carpal tunnel compliance after each step of flexor retinaculum release to the compliance with the FR intact at the proximal and distal levels of the carpal tunnel

The impact of incomplete FR release differed between the proximal and distal levels (Fig. 5). At the distal level, a minimum of 100 % TCL release was required to increase compliance significantly relative to compliance with the FR intact (*p* < 0.001). Subsequent DA release further increased compliance an additional 116 % although this increase did not represent a statistically significant gain relative to compliance with 100 % TCL release (*p* = 0.052). The compliance increase with AF transection was significantly greater than compliance with 100 % TCL release (*p* < 0.05) but not meaningfully different from the compliance with TCL and DA release (*p* > 0.05). At the proximal level, releasing 50 % of the TCL significantly increased compliance relative to compliance with the FR intact (*p* < 0.001). A 75 % TCL release increased compliance beyond the gain seen with 50 % TCL release (*p* < 0.05), and 100 % TCL release further increased CTC relative to CTC with 75 % TCL release (*p* < 0.05). DA release had only a minimal effect on CTC compared with 100 % TCL release at the proximal level (*p* = 0.987). However, complete FR release yielded a significant additional gain in compliance beyond that obtained with either 100 % TCL release or TCL + DA release (*p* < 0.05).

## Discussion

In this cadaver hand study, we investigated changes in the mechanical properties of the carpal tunnel with various degrees of FR release. We concluded that incremental FR release altered carpal tunnel structural mechanics as a function of compliance as calculated from the changes in carpal arch width. More specifically, the impact of FR release on carpal tunnel compliance was dependent on both the location within the carpal tunnel and the extent of FR release.

We found that complete FR transection significantly increased compliance by a factor of 4.3 at the distal level and 1.5 at the proximal level. This finding aligns with those of previous investigations [14, 16]. Previous studies have used different methodologies and outcome measures to evaluate the compliance features of the carpal tunnel, which limits our ability to directly compare the magnitude of compliance increase across studies. For example, Xiu et al. [16] defined compliance as the slope of the linear regression of the carpal arch width change as a function of outwardly applied forces of 2–10 N. Kim et al. [14] calculated compliance as the rate of change in the carpal arch area per change in carpal tunnel pressure applied via water infusion. In the current study, we used a constant 10 N force to stretch the carpal arch width as a means of evaluating the compliance changes of the carpal tunnel due to FR release. The compliance increase achieved by FR transection is one potential mechanism by which carpal tunnel release surgery can achieve median nerve decompression and symptom alleviation for patients who have carpal tunnel syndrome.

Our results show that complete TCL transection was required to significantly increase compliance at the distal level and to augment compliance relative to 50 or 75 % TCL release at the proximal level. This finding suggests that incomplete TCL release limits potential gains in structural flexibility of the carpal tunnel. Our findings differ somewhat from a prior study [17] that showed a gradual increase of bony carpal arch stretchability with progressive TCL sectioning. More specifically, the data reported in that study showed that 30 N loading led to a carpal arch distance change of 2.3 mm with the TCL intact, and distance changes of 2.7, 3.2, and 3.7 mm, respectively, when the TCL was progressively sectioned by 1/3, 2/3, and completely. The different pattern observed may be attributable to the differences in experimental protocols. Tengrootenhuysen et al. [17] used a threefold greater loading force and measured the change in carpal arch distance along an oblique orientation from the scaphoid tubercle at the proximal level to the hook of the hamate at the distal level. In contrast, in the present study, we measured along transverse axes at the proximal and distal levels. Our previous studies [16] have shown that the distal carpal tunnel at the hook of the hamate is relatively rigid, whereas the proximal carpal tunnel has greater flexibility. Mobility along the oblique axis is greater than that on the transverse axis at the proximal level but less than that at the distal level [18]. The greater distance changes along the oblique axis may be a function of the complex, three-dimensional motion of individual carpal bones [18].

TCL + DA release at the proximal level only minimally increased carpal tunnel compliance beyond the gain achieved with 100 % TCL release alone. In contrast, release of the TCL and DA at the distal level tripled compliance relative to the intact condition, whereas 100 % TCL release only doubled compliance. The small sample size may have contributed to the borderline significance of this result. This result points toward a trend that distal aponeurosis release has a greater effect on carpal tunnel compliance more distally within the carpal tunnel. Previous studies have demonstrated that DA release is required to decrease carpal tunnel pressure to below pathologic levels [19–22]. It is possible that the additive gains in compliance at the distal tunnel with DA release might explain the benefit of reduced carpal tunnel pressure after DA release. A previous study [23] did not report changes in carpal arch width after DA release; however, that study measured changes in carpal arch width without application of a loading force. Such a static measurement of carpal arch width captures only the resting state of the carpal tunnel structure rather than the intrinsic structural compliance of the carpal tunnel.

We observed a significant additional increase in compliance with AF release. At the proximal level, AF transection significantly increased compliance relative to both TCL + DA release and 100 % TCL release alone, whereas compliance at the distal level with complete FR (i.e., TCL + DA + AF) release was significantly greater than compliance with 100 % TCL release but only minimally greater than compliance with TCL + DA release. This difference suggests that AF release contributes to compliance gains to a greater extent at the proximal carpal tunnel than at the distal level. This additional gain in compliance with AF release helps explain previous findings that complete FR release, including AF release, significantly reduced carpal tunnel pressure beyond the level achieved with TCL + DA release alone [22]. That study also showed that AF release more robustly reduced pressure in the proximal carpal tunnel relative to more distal levels, which parallels our observation that AF release had a greater effect on compliance at the proximal level.

We found that incomplete TCL or FR release can mechanically constrain the carpal tunnel, causing residual focal compression that could contribute to median nerve ischemia. Clinically, incomplete FR release either proximally or distally is associated with persistent symptoms, including numbness, paresthesia, and difficulty manipulating small objects [4]. However, several clinical studies have suggested that release of the proximal FR may contribute to other postoperative complications that can delay return to work and resumption of activities of daily living [2]. These include pillar pain due to changes in carpal arch morphology that disrupt intercarpal articulations and grip weakness due to the FR’s reduced capacity to anchor the thenar and hypothenar muscles [24, 25]. Further research is needed to evaluate the pros and cons of DA and AF release. Our results suggest that DA release may be particularly beneficial for reducing mechanical constraints at the distal carpal tunnel.

We demonstrated that FR release increases local compliance more at the distal level of the carpal tunnel than at the proximal level. The carpal tunnel was found to be more rigid at the distal than the proximal level, irrespective of FR status, and a greater extent of FR release was required to effect a significant compliance change at the distal level than was needed at the proximal level. The relative inflexibility of the distal tunnel is a function of the differences in its structure compared with the proximal tunnel. The bones of the distal carpal arch form a rigid unit held in place by sturdy intercarpal ligaments that severely restrict movement [26]. In contrast, the proximal carpal arch is an intercalated segment with limitations on movement of its bones arising only from mechanical forces from their surrounding articulations [18]. Furthermore, the distal TCL has been shown to have a greater elastic modulus [27] and a lesser amount of strain [28] than the proximal TCL region. Clinically, the distal level of the carpal tunnel has been reported as a common site of median nerve entrapment [29, 30]. Complete FR release including the distal TCL + DA thus is particularly important for increasing compliance at this location.

There are several limitations to this study. First, a fixed FR release sequence was implemented. However, for each release step, there was no significant difference in the carpal arch width in the unloaded condition before and after loading, which suggests that there was no residual deformation of the carpal arches as a result of any specific transection or serial loading. Second, we had to use an artificial outward loading force applied to the carpal tunnel to investigate the structural properties of the carpal tunnel in the cadaveric specimens due to their lack of inherent physiologic or pathologic carpal tunnel pressure. Structural compliance is defined in the current study as the change in carpal arch width with respect to the 10 N loading force, which is different from the changes derived from the pressure-area relationship [14]. Finally, soft tissue around the FR, including the skin, muscles, and fascia, were dissected in the cadaveric specimens to allow digitization of the carpal bones and calculation of the carpal arch width. The effects of this soft tissue on carpal tunnel compliance are therefore not reflected in our results.

## Conclusions

In conclusion, we investigated changes in carpal tunnel compliance with incremental FR release and found that FR release increased the compliance of the carpal tunnel and had the greatest effect at the more rigid distal tunnel. Incomplete flexor retinaculum release without full transection of all three segments limits potential gains in carpal tunnel structural flexibility. Our study supports the benefit of complete flexor retinaculum release to maximize the effect of carpal tunnel release surgery to increase carpal tunnel compliance and thereby reduce carpal tunnel pressure and decompress the median nerve.

## Abbreviations

AF: antebrachial fascia
DA: distal aponeurosis
FR: flexor retinaculum
TCL: transverse carpal ligament
